# Sex- and age-dependent patterns of survival and breeding success in a long-lived endangered avian scavenger

**DOI:** 10.1038/srep40204

**Published:** 2017-01-11

**Authors:** Ana Sanz-Aguilar, Ainara Cortés-Avizanda, David Serrano, Guillermo Blanco, Olga Ceballos, Juan M. Grande, José L. Tella, José A. Donázar

**Affiliations:** 1 Department of Conservation Biology, Estación Biológica de Doñana, CSIC. Americo Vespucio s/n, Isla La Cartuja E-41092 Sevilla, Spain; 2 Population Ecology Group, Instituto Mediterráneo de Estudios Avanzados (CSIC-UIB), Miquel Marqués 21, E-07190 Esporles, Islas Baleares, Spain; 3 Ecology Area, Department of Applied Biology, Miguel Hernández University. Avda. de la Universidad s/n, E-03202 Elche, Alicante, Spain; 4 Infraestruturas de Portugal Biodiversity Chair CIBIO/InBIO. Centro de Investigação em Biodiversidade e Recursos Genéticos, Universidade do Porto. Campus Agrário de Vairão, 4485-661 Vairão, Portugal; 5 CEABN/InBio, Centro de Ecologia Aplicada “Professor Baeta Neves”, Instituto Superior de Agronomia, Universidade de Lisboa, Tapada da Ajuda, 1349-017 Lisboa, Portugal; 6 Department of Evolutionary Ecology, Museo Nacional de Ciencias Naturales, CSIC, José Gutiérrez Abascal 2, 28006 Madrid, Spain; 7 UGARRA, Avda. Carlos III 1, 31002, Pamplona, Spain; 8 Instituto de Ciencias de la Tierra y Ambientales de La Pampa, CONICET, Avda. Uruguay 151, 6300 Santa Rosa, La Pampa, Argentina; 9 Centro para el Estudio y Conservación de las Aves Rapaces en Argentina, Facultad de Ciencias Exactas y Naturales, Universidad Nacional de La Pampa, Avda. Uruguay 151, 6300 Santa Rosa, La Pampa, Argentina

## Abstract

In long-lived species, the age-, stage- and/or sex-dependent patterns of survival and reproduction determine the evolution of life history strategies, the shape of the reproductive value, and ultimately population dynamics. We evaluate the combined effects of age and sex in recruitment, breeder survival and breeding success of the globally endangered Egyptian vulture (*Neophron percnopterus*), using 31-years of exhaustive data on marked individuals in Spain. Mean age of first reproduction was 7-yrs for both sexes, but females showed an earlier median and a larger variance than males. We found an age-related improvement in breeding success at the population level responding to the selective appearance and disappearance of phenotypes of different quality but unrelated to within-individual aging effects. Old males (≥8 yrs) showed a higher survival than both young males (≤7 yrs) and females, these later in turn not showing aging effects. Evolutionary trade-offs between age of recruitment and fitness (probably related to costs of territory acquisition and defense) as well as human-related mortality may explain these findings. Sex- and age-related differences in foraging strategies and susceptibility to toxics could be behind the relatively low survival of females and young males, adding a new concern for the conservation of this endangered species.

Long-lived species typically present deferred maturity and their vital rates (survival and reproduction) are expected to change with age^1^. Age- and/or stage-dependent patterns of survival and reproduction determine the shape of the reproductive value function, which ultimately drives population dynamics^2^. Age-related improvements at the population level may occur as a consequence of the progressive selection of high-quality individuals (selection hyphotesis^1^,^3^), and/or by individual improvement due to acquisition of experience and performance in foraging, reproductive or migration skills, among others early in life (restraint and constraint hyphothesis^4^,^5^,^6^). Later, an age-related deterioration of performance and increased mortality probabilities due to senescence will be expected^7^. However, recent studies on long-lived birds indicate a very late life senescence (e.g. refs 8 and 9, which could also be masked by the selective disappearance of certain phenotypes among the older age-classes^1^).

Differences in age-dependent demographic parameters between sexes are also common in nature^10^,^11^, especially among species showing intense competition for mates (polygynous species)^10^,^12^, and/or body-sized dimorphic species with asymmetric parental roles^13^. However, sex-specific life-histories exist even in monogamous species with low sexual body-size dimorphism and similar parental investment in reproduction^9^,^11^. For example, recruitment (i.e., age of first breeding) differs between sexes in several bird species, including long-lived raptors^11^,^14^,^15^. In addition, sex-specific costs of reproduction^16^,^17^, susceptibility to human-related mortality^18^,^19^ or to diseases and parasites^20^ may be responsible for sex-biased survival. Regarding reproductive performance, it is difficult to separate the sex-specific contribution of partners of socially monogamous species, but a few studies found evidence of sex-differences in aging of reproductive traits (see review in ref. 9). For example, old male albatrosses (but not females) showed a sharp reproductive senescence from age 30 years onwards due to a lower foraging efficiency^21^.

Large avian scavengers (vultures and condors, Accipitridae and Cathartidae, respectively) are among the longest-lived vertebrates, with some individuals living over 40 years^22^. These species show delayed maturity such that individuals typically do not recruit into the breeding populations until they have at least acquired adult plumage patterns after several years of life^23^ (but see ref. 24). Consistent with this slow life-history strategy, large avian scavengers have low fecundity rates (commonly one chick per year). Existing demographic studies have focused on the description of basic parameters, with few studies addressing the combined contribution of age and sex^24^,^25^, which are essential for a robust diagnosis of population dynamics and a better understanding of life histories evolution^11^,^12^. In fact, among long-lived territorial species it is very difficult to achieve large sample sizes of known-age marked birds for long time periods (as that obtained for short lived and/or colonial species)^11^, which precludes an examination of how aging affects individual life-history strategies. For this reason, potential sex-specific trade-offs between recruitment, breeding success and/or survival remain understudied^26^. Filling these gaps in knowledge would also significantly benefit conservation; avian scavenger populations have declined abruptly worldwide, a trend considered one of the greatest examples of the current biodiversity crisis^27^. There is a general consensus that higher rates of non-natural mortalities, combined with the above-mentioned slow pace of life, are the main causes of population collapses^28^, and sex-specific asymmetries may also play a key role in these processes^18^.

Here, we take advantage of a long-term (31 year) monitoring program of a globally endangered avian scavenger, the Egyptian vulture (*Neophron percnopterus*), in North-Central Spain (Fig. 1) to study the basic demographic parameters of adult breeders (age of recruitment, breeding success and adult survival) in relation to sex and age. Since the parental roles of Egyptian vultures are roughly similar between sexes (see below), we expect similar parameters for males and females and/or age related improvements early in life (through acquisition of skills and experience or the (dis)appearance of different quality phenotypes) and/or late-in-life deteriorations due to senescence.

## Results

### Age of recruitment

Female and male Egyptian vultures showed a median age of first reproduction of 6 and 7 yrs, respectively (Fig. 2). Mean age of first reproduction was similar (6.88 yrs in females and 7.25 yrs in males, t = −0.645, DF = 39.243, p = 0.523) but the variance was significantly larger among females that among males (ranges: 3–14 yrs vs. 5–11 yrs, respectively; 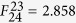, p = 0.014; Fig. 2).

### Breeding success

Regarding variations in breeding success with age, a first mixed modeling approach showed a tendency of Egyptian vultures to experience a higher probability of breeding successfully with the logarithm of age (estimate ± SE = 2.575 ± 1.013, Chi-square = 6.46, p = 0.001). In a second step, the within-subject centering method indicated that this increase in performance with age was due to the selective appearance and disappearance of certain individuals, being similar for both sexes, and not to improvement within individuals as they aged (Table 1). Importantly, age of first and last reproduction were positively correlated (rs = 0.76, p < 0.0001), so this demographic, between-individual effect seems to be explained by high-performing individuals recruiting and disappearing at higher ages than low-performing individuals. Thus, birds recruiting and disappearing at early ages had the lowest probability of breeding successfully, and birds recruiting and disappearing at late ages had the highest (Fig. 3).

### Survival

In survival analyses, the overall goodness-of-fit test of the Cormack-Jolly-Seber model of both female and male datasets was not statistically significant (Females: χ^2^ = 14.61, DF = 18, p = 0.69, males: χ^2^ = 0.01, DF = 8, p > 0.99). For females, several models describing age-dependent survival received similar support in terms of AICc, but no age structure reduced the AICc value of the constant model (Table 2). Mean female survival was 0.82 (95% CI: 0.74–0.88; Model 1, Table 2; Fig. 4). In contrast, model selection for males retained a model in which males ≤7 yrs and males ≥8 yrs had different survival probabilities: 0.61 (95% CI: 0.39–0.79) vs. 0.90 (95% CI: 0.83–0.94), respectively (Model 10, Table 2; Fig. 4). A model with a threshold at 8 yrs had a similar AICc and estimated a local survival probability of males ≤8 yrs and males ≥9 yrs of 0.70 (95% CI: 0.53–0.82) and 0.91 (95% CI: 0.84–0.95), respectively (Model 11, Table 2). Resighting probabilities for females and males were 0.90 (95% CI: 0.87–0.93) and 0.96 (95% CI: 0.94–0.98), respectively.

When combining the two datasets, a model including a sex effect but no age effects on survival was 1.31 units of AICc higher than a model without sex effects, indicating no clear sex effect on mean survival (Table 2 combined analyses). Mean survival for males (0.86, 95% CI: 0.79–0.90) was slightly higher than that of females, but CI overlapped (see above). However, when taking into account the best survival structures from the previous analyses, a model with female constant survival and male age-dependent survival (7yrs ≠ ≥8yrs) was clearly retained, reducing the AICc value by 5.77 units (Table 2 combined analyses). Old males showed a higher survival than females (Fig. 4).

## Discussion

Our results strengthen the importance of taking into account age and sex when studying life histories, even among monogamous species in which both sexes exhibit similar parental care^1^,^2^,^9^,^11^,^29^. Based on a unique long-term monitoring of know-age marked individuals in a population of a territorial vulture, we found that sex asymmetries constrained the age of recruitment and the local survival of breeding individuals, but not breeding success. Likewise, we detected a larger range of recruitment ages (especially among females) and a substantial mean delay in the acquisition of breeding territories compared to sexual maturity (especially among males). The density-dependent age of recruitment hypothesis predicts that individuals can advance the age of first reproduction when vacancies exist in the population, with recruitment serving as a buffering mechanism allowing persistence^30^. Our focal population is under clear decline linked to unnatural high mortalities^31^,^32^,^33^ but still showed a substantial delay in recruitment. Although a high plasticity in age of first reproduction is a common trait among long-lived species, early reproduction has been commonly associated with increased mortality and/or breeding costs^16^,^34^. In general, among long-lived species, a delay in the onset of reproduction beyond sexual maturity benefits individuals in terms of lifetime reproductive success, and may be an adaptive strategy^11^. Moreover, sex-specific differences in the optimal age of first reproduction have been described for numerous species^11^. For example, for two raptor species, the barn owl (*Tyto alba*) and the merlin (*Falco columbarius*), females benefited more from earlier onset of reproduction than males^11^. Accordingly, our results also suggest that a tradeoff between survival and early recruitment could explain the delayed recruitment of males observed in this declining population.

As expected under the general evidence of age-related improvements in reproductive performance in birds (e.g. selection, restraint and constraint hypothesis^1^), we found that Egyptian vultures increased their probability of breeding successfully with age, but only at the population level. Our analyses allowed to separate the contributions of between and within-individual age effects on breeding success indicating that demographic mechanisms, rather than ontogenetic processes (restraint and constraint hypothesis), were behind the observed age-related improvement of reproductive success^29^,^35^. Thus, low-quality individuals with lower reproductive performance would appear (i.e., recruit) and disappear from the breeding population at earlier ages (selection hypothesis^4^) and high quality individuals would start breeding and disappear at later ages. We cannot overlook that individual improvements may be related to the potential acquisition of experience in reproductive skills and/or other indirectly associated activities, such as foraging and territory defense^5^,^6^, and that these effects might arise with larger sample sizes. Egyptian vultures likely gain experience during their long pre-reproductive years before becoming breeders at quite advanced ages^11^.

Species with monogamous mating systems generally lack sex bias in mortality (see a review in ref. 9). However, sex-specific susceptibility to human-related mortality linked to behavioral mechanisms^19^ or small sex-specific differences in reproductive investment may be behind sex-biased survival, being especially important for inexperienced and/or young individuals^16^,^17^. Here, we detected different patterns of survival among sexes. Namely, female breeders did not show the aging effects of males. Old male breeders had comparatively higher local survival (0.90) than young ones (0.61), with this last estimate being very similar to a previous estimate for non-breeding 5-year-old birds in the Ebro population in northern Spain (0.60)^25^. These low survival rates may be explained by the fact that birds on the verge of adulthood may also make a substantial investment in prospecting, thus being exposed to greater risks of unnatural mortality^25^ (e.g. human persecution, accidents). Accordingly, we could hypothesize that young breeding males may maintain exploratory behaviors and be exposed to higher anthropogenic mortality risks. Our results could also indicate a male-specific cost associated with early reproduction. Male and female Egyptian vultures are almost monomorphic in size but males have more intense secondary sexual characters (e.g., the color of the face, linked to carotenoid acquisition^36^). Also, males perform major active territory defense (e.g. displaying complex undulating flights) against conspecific and heterospecific intruders (authors unpublished), while females would select mates without incurring in such costs. Thus, our results suggest that females may recruit at earlier ages than males without survival costs, and this would explain the larger observed variance in female age of first reproduction^11^. Later in life, a decrease in survival (or reproductive success) at advanced ages (i.e., senescence^9^) was not detected for any sex. It is possible that the study period was relatively short with respect to the species’ potential longevity (captive birds survived up to 37 years^22^), or that sample size for very old individuals was small and senescence in long-lived birds has usually been detected very late in life^8^,^21^. It seems also reasonable that the relatively high rates of mortality of breeding adults detected here would preclude the appearance or detectability of senescence traits in our study population.

Importantly, female Egyptian vultures in our study population showed an alarmingly low local survival in comparison with the survival rates estimated for adults breeding in other European populations and other large body-sized scavengers^8^,^37^,^38^. Higher rates of female mortality as that found here have been claimed as the main factor leading to male-skewed adult sex-ratios in many bird species with balanced sex-ratios at birt^39^. As stated before, asymmetric mortalities between sexes may occur due to sex-specific costs of reproduction^17^ but also to sex-specific differences in foraging strategies and habitat selection, as occurs in other avian species^19^,^39^,^40^.

From a large-scale perspective, the survival of trans-Saharan migrant species is influenced by the ecological conditions in wintering areas^25^,^41^. Although data are scarce, it appears that both male and female Egyptian vultures have similar home ranges during the breeding and wintering period in African Sahel regions^42^,^43^,^44^. However, different and decisive factors may be operating at a smaller scale. As occurs in other top scavenger species, such as in Andean condors (*Vultur gryphus*), the differential exploitation of space in terms of microhabitat between sexes may explain the observed patterns^41^,^45^. In our case study, the females may more frequently exploit predictable food sources, such as dumps and supplementary feeding stations, than males (ref. 44, authors’ unpublished data). This could also be the case for young and inexperienced males. By foraging on human-delivered waste individuals may be more exposed to associated risks (e.g. poisons, pathogens and/or toxic veterinary pharmaceutical^46^). In the Ebro Valley, although data are scarce, from a total of 13 known-sex Egyptian vultures found dead by poison, 9 (69%) were females (authors’ own data).

Overall, our findings present an approach to understand the evolution of sex-specific life-history strategies in long-lived birds while also posing concerns facing the conservation of this globally threatened species. Susceptibility to anthropogenic factors is one of the main sources of vulnerability of wildlife populations^47^. In the case of the threatened Egyptian vulture, given that anthropogenic mortality continues to be high in some European regions, the recovery of its populations is challenging^28^. Moreover, the female-skewed mortality that we found here adds concern for the conservation of this globally endangered species. In fact, if differences in survival between sexes would be ignored (e.g. by using the mean survival estimate (0.84, Model 1, Table 2 combined analyses)) the survival of breeding females would be overestimated resulting in biased and too optimistic predictions of population viability analyses. Unbalanced sex ratios in the adult fraction of the population would lead to a reduction in effective population size and ultimately reduce population viability in the medium and long term, as predicted for other large avian scavengers such as the Andean Condor^18^. The conservation of carrion-eaters in the Old World has focused on the importance of tackling direct and indirect deaths from persecution, mainly due to the ingestion of toxics^27^. Our study also emphasizes that further studies deepening our knowledge of the potential age and/or sex-specific differences in habitat use and risks of toxicity associated with the use of predictable food sources (dumps and supplementary feeding stations) are urgently needed^48^.

## Methods

### Study species

The Egyptian vulture is a globally endangered medium-sized scavenger (weighing around 2 kg) living in dry and mountain biomes of southern Europe, Asia and Africa. Formerly very abundant, the global population of Egyptian vultures has experienced a severe decline throughout its range^49^. The Spanish population comprises ca. 80% of the European population and in some regions has seen a concerning decline in the last two decades^31^. Egyptian vultures are monogamous and highly philopatric to their breeding territories^31^,^50^. Although both sexes are indistinguishable in plumage, females are slightly larger and ~10–15% heavier than males. Females typically produce 2 eggs per clutch, which are incubated by both parents, which also share parental investment during chick rearing^51^. This highly opportunistic species forages on small wild prey and on carcasses of small and medium-sized animals^32^,^50^. Continental Western European populations of Egyptian vultures spend the wintering season (and sometimes their first year of life) in the sub-Saharan Sahel region^42^. Pre-breeder survival varies with age: it increases up to 4 yrs of age and then decreases at 5 yrs of age when birds acquire adult plumage, abandon the communal lifestyle of juveniles, and usually begin to look for a breeding territory^25^. Little is known, however, about age- and sex-dependent recruitment patterns, breeding success and breeder survival^28^,^37^.

### Study areas and field procedures

**Figure 1:**
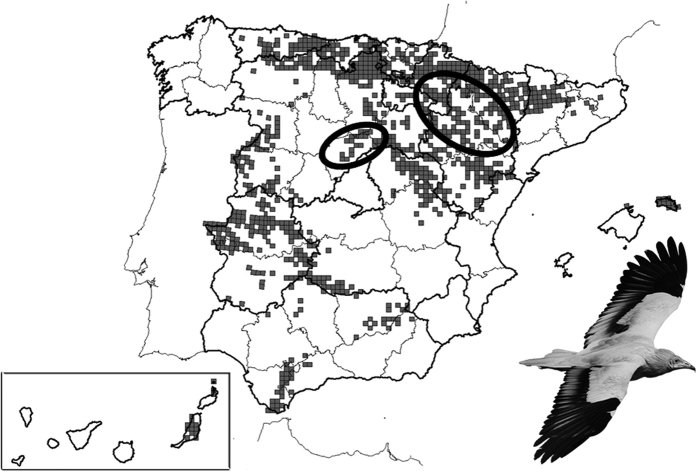
Egyptian vulture breeding distribution in Spain (grey dots) and the two study areas shown with an ellipse. Note that this figure has been modified from Donázar (2004)^60^ with the approval of SEO/Birdlife. Map Credit: J. C. del Moral Photo Credit Egyptian vulture: M. de la Riva.

**Figure 2:**
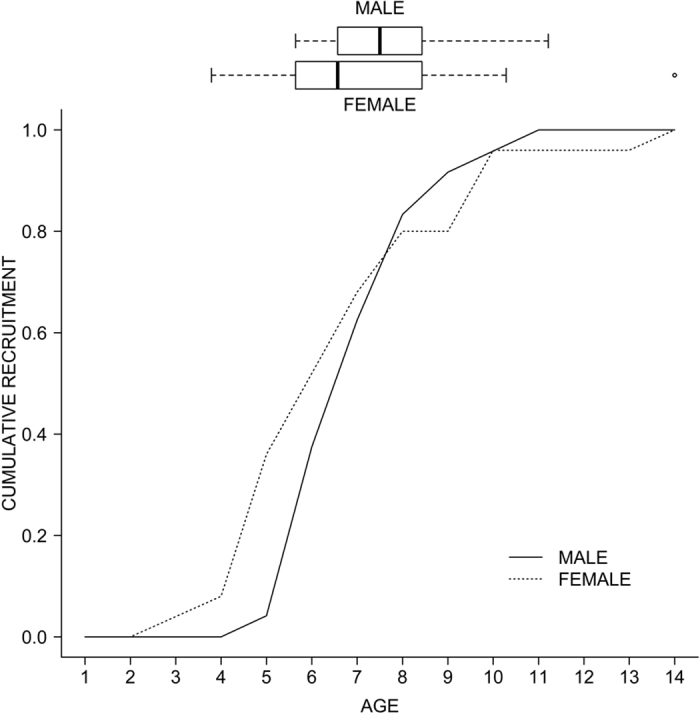
Median and cumulative observed age of recruitment of female (n = 25) and male (n = 24) Egyptian vultures.

**Figure 3:**
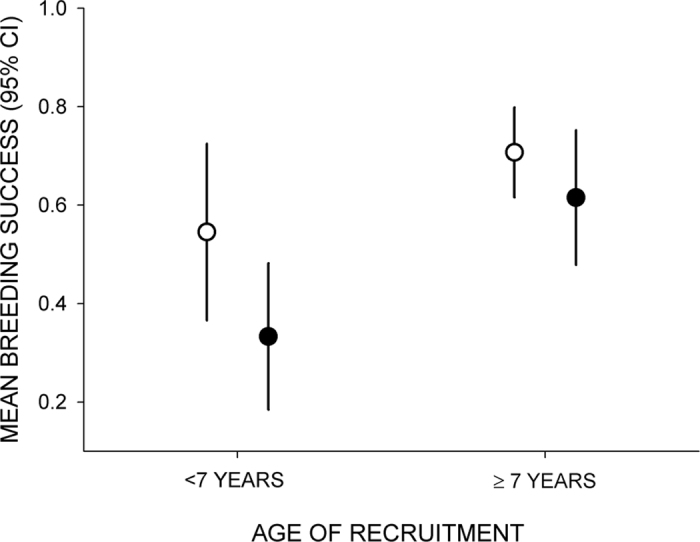
Breeding success probabilities of female (black points) and male (white dot) Egyptian vultures aged <7 and ≥7 years.

**Figure 4:**
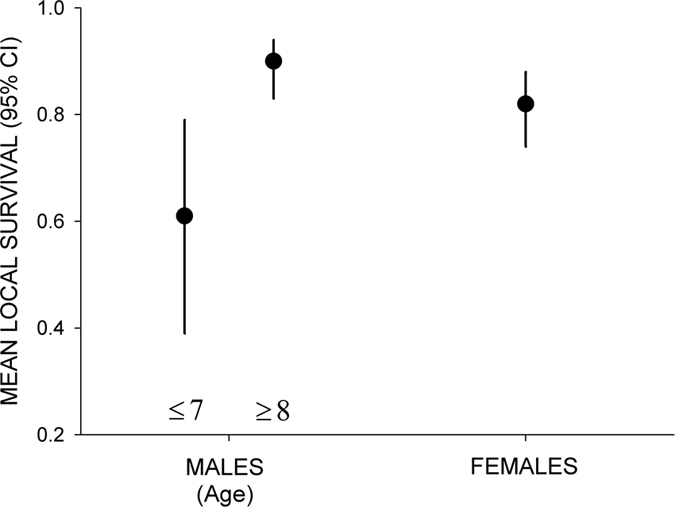
Local survival probabilities of female (n = 31) and male (n = 30) Egyptian vultures (estimates from Model 3, Table 2 **Model Survival Structure (combined analyses)).**

The research was performed in North-Central Spain (Fig. 1). The monitoring program started in 1986, searching for territories and ringing chicks, and an exhaustive monitoring of breeding birds at territories in the Ebro Valley (19000 km^2^) has been carried out since 1992. Additionally, in 2003, the monitoring program extended to Segovia (1800 km^2^) and additional territories and breeding birds were included in the monitoring of the population that has continued to date (2016). Overall, during the study period, 1018 Egyptian vultures of known age have been captured and marked with both aluminum and plastic rings allowing their long-distance identification (918 nestlings in their nests and 100 immature birds using cannon nets). Moreover, 70% of them were sexed with molecular techniques allowing the robust determination of an unbiased secondary sex-ratio (N = 717 fledglings, 0.53 females, 0.47 males, Binomial test p = 0.1167).

We surveyed most breeding territories within the study area (including active and abandoned territories^31^) at least three times per breeding season to collect data on territory occupancy and breeding success, to identify ringed breeders present at territories and to ring and sample fledglings (see details in ref. 25). From 1992 to 2016, 61 marked individuals (31 females and 30 males, representing 6% of the 1018 marked individuals of known age) have been detected breeding in the focal study area (N = 59) and adjacent territories in Central Spain (N = 2) located between the two exhaustively monitored areas (Fig. 1). Our monitoring allowed us to record the precise age of first reproduction for 49 individuals (25 females and 24 males). The remaining 12 birds corresponded to individuals of an uncertain age of recruitment observed breeding for the first time in territories not accurately monitored the year before (e.g., breeding birds at new territories that could have gone unnoticed or territories that failed in an early reproductive stage without time for identifying breeding adults). Maximum lifespan detected was 24 (N = 1) and 21 (N = 1) years for males and females, respectively (with individuals still alive at the end of the study, thus representing a minimum estimate of maximum lifespan).

### Analytical procedures

Despite the intense monitoring effort, it was not always easy to ascertain if the breeders associated with each territory were marked and/or to detect early reproductive failures. Therefore, uncertain data (see above) were discarded for recruitment tests. Sex differences in the mean and variance of age of first reproduction were analyzed by means of a t-test and F-test, respectively.

We used Generalized Linear Mixed Models (GLMM)^51^ using the package lme4^52^ in R v. 3.1.3^53^ to assess the effects of age on breeding success using de data of 226 breeding events (see detailed sample sizes in Table S1). The response variable was whether or not the individual bred successfully (binomial mixed model with logit link function), while bird identity and year were fitted as random terms to account for multiple measurements of the same individual and temporal heterogeneity, respectively. We initially explored if variation in breeding success could be explained by the linear, logarithmic or quadratic effects of age. This approach, however, does not allow for distinguishing whether potential variation in breeding success over age is due to within-individual changes (improvement, senescence, terminal effects) or to between-individual heterogeneity (the selective appearance or disappearance of certain phenotypes)^29^. Within-subject centering (*sensu*^54^) is a statistical method to do so, and has been shown to be useful in non-experimental situations^55^. We applied this technique by fitting mixed-effects models with the following structure:


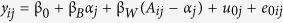


where *β*_B_ is the between-individual effect of age of first or last reproduction (for testing the selective appearance or disappearance of individuals, respectively), *β*_W_ is the within-individual effect of delta age (the age at which the breeding success of each individual was measured minus individual age of first or last reproduction), *u*_0*j*_ is the random intercept and *e*_0*ij*_ the residual variance.

Survival probabilities were modeled by means of multi-event capture-recapture models^56^ allowing for the combination of different types of data (on individual monitoring of known-age breeders (252 observations) and on territory monitoring) to improve parameter estimation and to differentiate true absences from detection failures^56^. The multi-event framework distinguishes what can be observed in the field (the events coded in the encounter histories) from the underlying biological states of the individuals, which must be inferred^57^. Our model included two biological states: locally alive (coded A) and locally dead (coded D). Encounter histories were coded using three different events (see below). Each row of encounter histories belonged to a different individual and each column referred to the individuals’ age (from the youngest to the oldest age that individuals could reach at the end of the study) instead of the classical individual histories by year. The three events used were:

Event ‘0’ was used to indicate that: (I) at a particular age the individual had not yet been detected breeding (i.e., events before recruitment); (II) at a particular age after recruitment, an individual was not breeding in its territory, with its territory empty or occupied by different breeders; (III) the individual was recovered dead (i.e., the vulture did not reach this particular age); and (IV) the individual could not reach this particular age class due to the length of the study. These cases were not taken into account in the analyses (i.e., the encounter histories were right censored, using a ‘−1’ code in an additional column, see details in ref. 58. Consequently, the information of ‘0’ event only refers to a locally dead state.

Event ‘1’ was used to indicate that a vulture was observed breeding at a particular age. Consequently, this event only refers to a locally alive state.

Event ‘2’ was used to indicate that a vulture was not observed at a particular age in a territory not accurately monitored this year. Consequently, this event can refer to both alive and dead states.

The multievent model estimates the probabilities of transition between the states (survival ф and mortality 1 − ф probabilities, matrix 1) and the probability of resighting (*p*, matrix 2) that relates the observed events with the individual state. This model assumed that when a territory was properly prospected (events 0 and 1) the state of the individual (locally alive or dead) was known with certitude. Consequently, the resighting probability, *p,* is the probability of a proper prospection of territories and *1-p* represent detection/prospection failure (event 2). However, note that survival and mortality probabilities estimated in this way must be considered local/apparent (i.e., they do not allow for distinguishing between mortality and emigration).


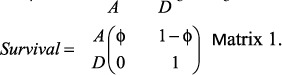



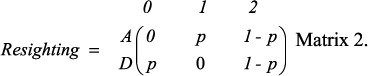


Capture–recapture analysis began with the assessment of the goodness-of-fit (GOF) of the Cormack-Jolly-Seber model to the data using program U-CARE 2.3.2^58^. Parameters were estimated simultaneously by maximum likelihood using program E-SURGE 1.6.3^58^. To avoid overparameterized models, we first built models for males and females separately, and all models considered a single resighting probability parameter. We tested the effects of age on survival by considering no age effects, full age effects, linear, quadratic and logarithmic curves, and different age structures including an arbitrary threshold (from 3 to 12 yrs old) to differentiate young and old breeders. Finally, we combined both datasets to test for between-sex differences. Model selection was based on Akaike’s Information Criterion adjusted for the effective sample size, AICc^59^. Models within 2 points of AICc were considered equivalent.

#### Ethic statements

Capture banding and monitoring of Egyptian vultures were conducted under permits and following the protocols approved by the competent Regional Governments of Navarre, Aragón and Castilla-León and following the protocols approved by the Ethic Committee of CSIC (CEBA-EBD-12-56), in accordance with the approved guidelines.

## Additional Information

**How to cite this article**: Sanz-Aguilar, A. *et al*. Sex- and age-dependent patterns of survival and breeding success in a long-lived endangered avian scavenger. *Sci. Rep.* **7**, 40204; doi: 10.1038/srep40204 (2017).

**Publisher's note:** Springer Nature remains neutral with regard to jurisdictional claims in published maps and institutional affiliations.

## Supplementary Material

Supplementary Information

## Figures and Tables

**Table 1:** Binomial mixed models to distinguish within- and between-individual age effects on breeding success of Egyptian vultures.

	Fixed effect	Estimate	SE	Chi-square	p-value
*Selective appearance*					
Minimum model	Intercept	−3.325	1.271		
	Delta age (*β*_W_)	0.254	0.163	2.43	0.12
	Age of first reproduction (*β*_B_)	1.732	0.638	7.37	0.007
	*R*^*2*^	0.22			
Rejected terms	Delta Age × Age of first reproduction	0.195	0.626	0.10	0.76
	Sex (Males)	0.925	2.643	1.25	0.26
	Sex (Males) × Delta age	−0.058	0.340	0.03	0.86
	Sex (Males) × Age of first reproduction	−0.242	1.330	0.03	0.86
*Selective dissapearance*					
Minimum model	Intercept	−3.146	1.521		
	Delta age (*β*_W_)	−0.076	0.221	0.12	0.73
	Age of last reproduction (*β*_B_)	1.477	0.653	5.12	0.024
	*R*^*2*^	0.21			
Rejected terms	Delta Age × Age of last reproduction	0.197	0.551	0.13	0.72
	Sex (Males)	3.752	3.328	0.73	0.39
	Sex (Males) × Delta age	−0.356	0.498	0.51	0.47
	Sex (Males) × Age of last reproduction	−1.393	1.431	0.95	0.33

The selective appearance of phenotypes (between-individual effects) was tested by including the natural logarithm of age of first reproduction into the models, while individual changes with age (within-individual effects) was tested by subtracting individual age of first reproduction from individual age when each breeding event was recorded (logarithmized delta age). The selective disappearance of individuals was tested in a similar way, but replacing age of first reproduction with age of last reproduction. The minimum model is the minimum retained model necessary to separate within- and between-individual effects^53^. Variance explained by each model (conditional *R*^*2*^) is shown^61^.

**Table 2:** Modeling age-dependent survival of Egyptian vultures.

Model	Survival Structure	Females			Males		
		np	AICc	ΔAICc	np	AICc	ΔAICc
1	Constant	**2**	**390.30**	**0**	2	244.63	7.08
2	Age	16	411.66	21.36	17	304.32	66.77
3	A	3	391.68	1.38	3	244.19	6.63
4	A^2^	4	393.27	2.97	4	239.24	1.69
5	Log(A)	3	391.95	1.65	3	241.56	4.00
6	[3 yrs] ≠ [≥4 yrs]	3	391.98	1.67	—	—	—
7	[≤4 yrs] ≠ [≥5 yrs]	3	391.99	1.69	—	—	—
8	[≤5 yrs] ≠ [≥6 yrs]	3	392.29	1.99	3	246.39	8.84
9	[≤6 yrs] ≠ [≥7 yrs]	3	392.27	1.97	3	242.38	4.83
10	[≤7 yrs] ≠ [≥8 yrs]	3	392.19	1.89	**3**	**237.55**	**0**
11	[≤8 yrs] ≠ [≥9 yrs]	3	391.53	1.23	3	238.25	0.69
12	[≤9 yrs] ≠ [≥10 yrs]	3	392.38	2.08	3	241.02	3.47
13	[≤10 yrs] ≠ [≥11 yrs]	3	392.36	2.06	3	243.29	5.73
14	[≤11 yrs] ≠ [≥12 yrs]	3	392.37	2.07	3	244.73	7.18
**Model**	**Survival Structure (combined analysis)**				**np**	**AICc**	**ΔAICc**
1	Constant				3	633.59	5.77
2	Sex				4	634.90	7.08
**3**	**females (Constant)/males ([≤7 yrs]** **≠** **[≥8 yrs])**				**5**	**627.82**	**0**

Model Survival Structure: Separate analyses by sex and Model Survival Structure (combined analyses). Notation: np = number of parameters; AIC = Akaike information criterion corrected for small sample size; ΔAICc = AICc difference between the current model and that with the lowest AICc value. Model notation: Age = full differences among age classes; A = lineal effect of age; A^2^ = quadratic effect of age; Log(A) = logarithmic effect of age.
