# Supplementary material for: Sex- and age-dependent patterns of survival and breeding success in a long-lived endangered avian scavenger
Source: Sci Rep. 2017 Jan 11;7:40204. doi: 10.1038/srep40204 (PMC5225485; doi:10.1038/srep40204)
Supplement: Supplementary Information [file srep40204-s1.pdf]

Supplementary Information

Sex- and age-dependent patterns of survival and breeding success in a long-lived endangered avian scavenger

Ana Sanz-Aguilar[1,2,3°], Ainara Cortés-Avizanda [1,4,5*°], David Serrano[1], Guillermo Blanco[6], Olga Ceballos[7] , Juan M. Grande[8,9], José L. Tella[1] and José A. Donázar[1]

[1] *Department of Conservation Biology, Estación Biológica de Doñana, CSIC. Americo Vespucio s/n, E-41092 Sevilla, Spain.*

[2] *Population Ecology Group, Instituto Mediterráneo de Estudios Avanzados (CSIC-UIB), Miquel Marqués 21, E-07190 Esporles, Islas Baleares, Spain.*

[3] *Ecology Area, Department of Applied Biology, Miguel Hernández University. Avda. de la Universidad s/n, E-03202 Elche, Alicante, Spain.*

[4] *Infraestruturas de Portugal Biodiversity Chair CIBIO/InBIO. Centro de Investigação em Biodiversidade e Recursos Genéticos, Universidade do Porto. Campus Agrário de Vairão, 4485-661 Vairão, Portugal.*

[5] *CEABN/InBio, Centro de Ecologia Aplicada "Professor Baeta Neves", Instituto Superior de Agronomia, Universidade de Lisboa, Tapada da Ajuda, 1349-017 Lisboa, Portugal.*

[6] *Department of Evolutionary Ecology, Museo Nacional de Ciencias Naturales, CSIC, José Gutiérrez Abascal 2, 28006 Madrid, Spain.*

[7] *UGARRA, Avda. Carlos III 1, 31002, Pamplona, Spain.*

[8] *Instituto de Ciencias de la Tierra y Ambientales de La Pampa, CONICET,Avda. Uruguay 151, 6300 Santa Rosa, La Pampa, Argentina.*

[9] *Centro para el Estudio y Conservación de las Aves Rapaces en Argentina, Facultad de Ciencias Exactas y Naturales, Universidad Nacional de La Pampa, Avda. Uruguay 151, 6300 Santa Rosa, La Pampa, Argentina.*

*Corresponding Author: cortesavizanda@gmail.com; ainara@ebd.csic.es

° Both authors contributed equally to this work

Table S1. Sample sizes for analyses performed to distinguish within- and between-individual age effects on breeding success of Egyptian vultures. *Age* shows the number of breeding events recorded per age-class, while *Years* shows the number of individuals disaggregated by the number of years of monitoring. Sample sizes are shown for individuals whose age of first (Appearance) and last (Disappearance) breeding was known.

Age/Years	1	2	3	4	5	6	7	8	9	10	11	12	13	14	15	16	17	18	19	20	21	22	23	24
**Appearance**																								
Age																								
Males	0	0	0	0	3	9	9	13	12	12	14	9	9	7	5	5	5	5	4	3	3	2	2	1
Females	0	0	1	2	6	9	14	12	10	13	10	7	3	3	2	1	0	0	0	0	1	0	0	0
Years																								
Males	10	1	4	3	4	3	1	1	1	0	1	0	0	0	1	0	0	0	0	0	0	0	0	0
Females	8	3	5	4	2	5	3	0	0	0	0	0	0	0	0	0	0	0	0	0	0	0	0	0
**Disappearance**																								
Age																								
Males	0	0	0	0	2	9	8	7	6	7	7	3	5	4	3	3	3	3	3	2	2	1	1	0
Females	0	0	1	2	5	8	11	11	8	10	8	6	2	2	1	0	0	0	0	0	0	0	0	0
Years																								
Males	9	0	4	1	3	2	0	1	1	0	0	0	0	0	1	0	0	0	0	0	0	0	0	0
Females	6	3	5	4	1	3	2	0	0	0	0	0	0	0	0	0	0	0	0	0	0	0	0	0
